# Bridging mesoscopic and microscopic scales in multiple sclerosis: Post mortem brain block multi-contrast 9.4T MRI and histology quantification

**DOI:** 10.1016/j.xpro.2025.104203

**Published:** 2025-11-17

**Authors:** Dimitrios G. Gkotsoulias, Lukas Schönenberger, Jochen Leupold, Ilaria Callegari, Erik Bahn, Christine Stadelmann, Dominik von Elverfeldt, Valerij G. Kiselev, Matthias Weigel, Cristina Granziera

**Affiliations:** 1 Research Center for Clinical Neuroimmunology and Neuroscience Basel (RC2NB), University of Basel, Basel, Switzerland; 2 Translational Imaging in Neurology Basel, Department of Biomedical Engineering, Faculty of Medicine, University of Basel, Allschwil, Switzerland; 3 Department of Neurology, University Hospital Basel, Basel, Switzerland; 4 Division of Medical Physics, Department of Diagnostic and Interventional Radiology, University Medical Center Freiburg, Faculty of Medicine, University of Freiburg, Freiburg, Germany; 5 Department of Neuropathology, University Medical Center Göttingen, Göttingen, Germany; 6 Division of Radiological Physics, Department of Radiology, University Hospital Basel, Basel, Switzerland; 7 Department of Neurophysics, Max Planck Institute for Human Cognitive and Brain Sciences, Leipzig, Germany

**Keywords:** Health Sciences, microscopy, Neuroscience

## Abstract

Combining *ex vivo* MRI mesoscopic and histopathology microscopic assessments of tissue microstructure forms a key approach in multiple sclerosis (MS) research for elucidating disease pathogenesis. We present a protocol that enables reproducible, scalable, and robust integration of MRI and histology data in MS brains. We describe steps for (1) obtaining high-resolution multi-contrast 9.4T MRI of postmortem brain blocks and (2) the usage of a custom, open-source image processing interface for robust histology-MRI registration and semi-quantitative mapping of myelin density, microglia, and cell count.

## Highlights

• Procedures for acquisition of 9.4T MRI on postmortem brain blocks
• Instructions for deriving myelin, microglia, and cell density maps from IHC
• Instructions for co-registration of MRI contrasts and histology-derived maps

## Abstract (editor highlights)

Publisher’s note: Undertaking any experimental protocol requires adherence to local institutional guidelines for laboratory safety and ethics.

## Graphical abstract

**Figure:**
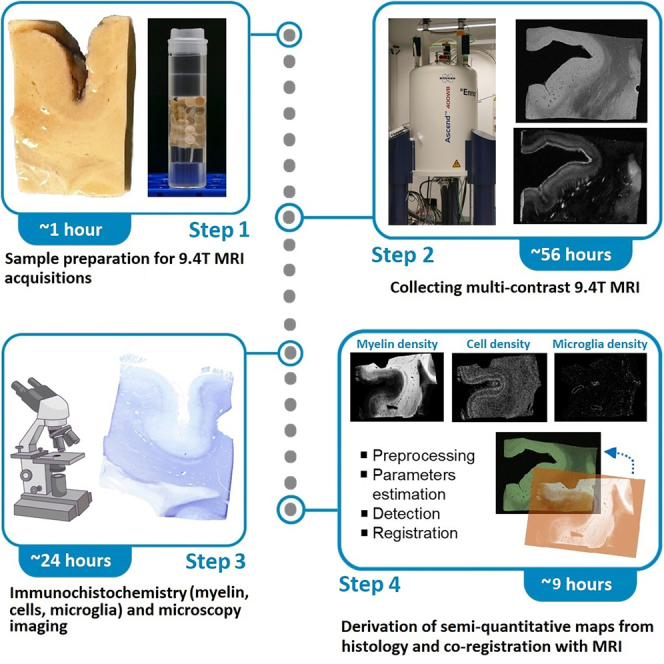

## Before you begin

The protocol below describes the specific steps for obtaining artifact free, high resolution multi-contrast 9.4T MRI of brain blocks, of approximate size of 2 × 2 × 1 cm, on a Bruker spectrometer. Subsequently, it provides detailed instructions on how to use a custom, semi-automated software to derive maps of myelin density, microglia density and cell density and register them to the MRI space.

To yield the most reliable study results, maintaining the lowest possible interval between the time of death and brain tissue fixation is crucial. Additionally, the conditions of extraction/fixation should be precisely documented. Tissue fixation time shall be documented and, ideally, standardized across all tissue blocks, as fixation has been shown to affect T1, T2 and T2∗ relaxation properties of brain tissue, and hence, all derivative contrasts that are based on those.^1^ If tissue dehydration is suspected, one shall consider Phosphate Saline Buffer (PBS) immersion of the tissue blocks, as proposed in previous works^2^ -however, the actual hydration of the tissue achieved with this methodology is not controllable and can lead to further inconsistencies.^3^ Documenting the reason of death -regardless of relevance to MS- which might alter the quality of brain tissue, for example, cerebral infarctions or trauma, is also essential.

**Figure 1:**
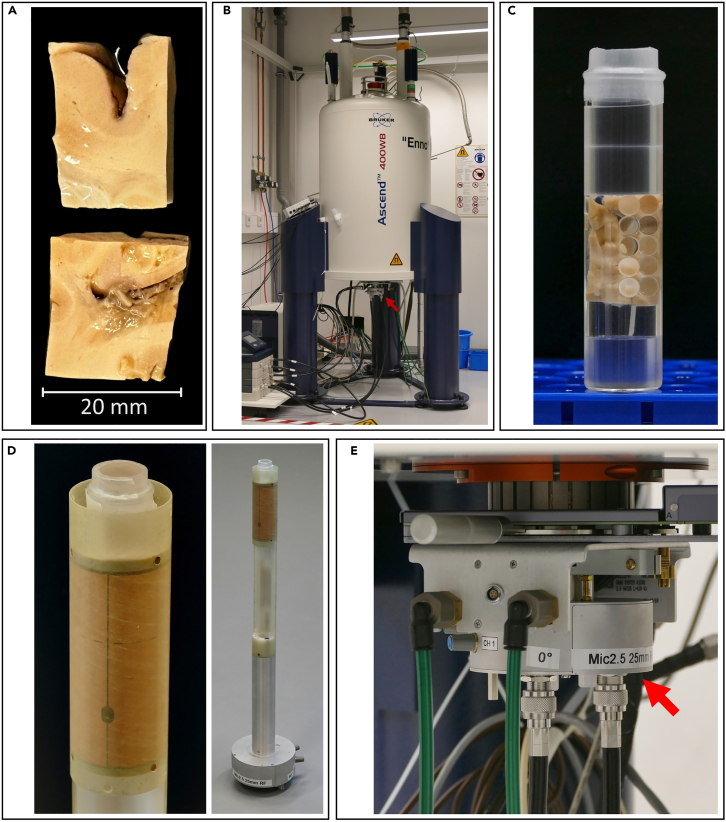
Tissue and MRI measurements setup used in our experiments (A) Indicative brain blocks containing regions of interest (ROIs) dissected post-mortem from donated brains of patients with MS. (B) The Bruker Avance Neo 400 WB spectrometer. (C) Image of the brain samples immersed in perfluoropolyether and stabilized with PMMA spheres and PMMA plugs. (D) Image of the specimen placed into the 25 mm birdcage RF receiver coil provided by the vendor and (E) mounted into the spectrometer (red arrows indicating the mounted coil base -see also B and D).

Obtaining full brain scans for the identification of lesions, their localization in the tissue and the eventual dissection of brain blocks containing regions of interest (ROIs) in size allowing for 9.4T brain scans (Figure 1A), according to the methodology explained in *Galbusera R. et al. 2022*,^4^ is recommended. For the **extraction of brain blocks** with precision to the ROI location, the user shall follow the methodology described in *Galbusera R. et al. 2022*.^4^

The software was developed and tested on ***Ubuntu 24.04.2 LTS, Release: 24.04*** operating system (OS), on a computer with 64bit CPU architecture (13th Gen Intel Core i7-13800H) and 64GB RAM. Please make sure to use the same, or newer OS version, and a system with comparable computational power (or higher).

### Innovation statement

This protocol provides details on a robust and extendable pipeline for the integration of ex vivo MRI and histology in post-mortem human brain research. It standardizes the preparation and acquisition steps for multi-contrast 9.4T MRI on fixed brain tissue blocks, ensuring high-quality and reproducible imaging data. The pipeline incorporates custom, open-source software interface that enables semi-automated derivation of myelin, microglia, and cell density maps from histology -with specificity for the major stains used in the characterization of multiple sclerosis lesions. Furthermore, on the same interface a dedicated co-registration framework for histology-derived maps, segmentations, and high-resolution MRI contrasts is included. This integrative workflow bridges the gap between MRI (mesoscopic level) and histology (microscopic level), facilitating a transparent and reproducible comparison of microstructural and cellular features.

### Institutional permissions

The brains were obtained from the German MS Brain Bank of the Competence Network Multiple Sclerosis (KKNMS): (https://www.kompetenznetz-multiplesklerose.de/patienteninformation/#ms-brain-bank). Sectioning of the brains, utilization of full-brain MRI and brain block MRI, as well as histological analyses, were approved by the ethical review committee of the University Medical Center Göttingen.

We remind readers that appropriate approvals from their respective institutions or ethical review committees must be obtained prior to conducting experiments such as those described in this protocol.

## Key resources table

**Table:** 

REAGENT or RESOURCE	SOURCE	IDENTIFIER
**Chemicals, peptides and recombinant proteins**		

Formaldehyde solution 4%, buffered, pH 6.9	Sigma-Aldrich	100496
PBS (phosphate-buffered saline)	Sigma-Aldrich	P4417

**Biological samples**		

Blocks from brains of adult multiple sclerosis patients	German MS Brain Bank of the Competence Network Multiple Sclerosis (KKNMS), University of Göttingen	NA

**Software and algorithms**		

OMERO v.5.6.3	University of Dundee & Open Microscopy Environment (OME)	https://www.openmicroscopy.org/omero/downloads/
QuPath v.0.5.1	Bankhead, P. et al. QuPath: Open-source software for digital pathology image analysis. (2017). Scientific Reports. *https://doi.org/10.1038/s41598-017-17204-5*	https://github.com/qupath/qupath/releases/tag/v0.5.1
QuPath’s OMERO extension v.0.1.0-rc5	See above	https://github.com/qupath/qupath-extension-omero
Python 3.11	Python Software Foundation	https://www.python.org/downloads/
Miniconda	Anaconda, Inc	https://www.anaconda.com/docs/getting-started/miniconda/main
Required Python Packages	See *requirements.txt* files for each processing pipeline	https://github.com/LukasSchoenenberger/IHC-Analysis-Pipeline

**Other**		

Avance Neo 400 WB spectrometer	Bruker	https://www.bruker.com/en/products-and-solutions/mr/nmr/avance-nmr-spectrometer.html
BX41 Optical microscope	Olympus/Evident	https://evidentscientific.com/en/upright/bx-microscopes
Slideview VS200 Slide Scanner	Olympus/Evident	https://evidentscientific.com/en/products/slide-scanners/vs200

## Step-by-step method details

### Sample preparation for 9.4T MRI acquisitions

**Timing: 1 h**

This step allows for proper placement of the specimen in the vendor’s 9.4T MRI system and ensures the quality of the subsequent acquisition. Please follow the instructions adapted to the MRI spectrometer vendor in each case. In our study, a **Bruker Avance Neo 400 WB spectrometer** was employed (Figure 1B). See also problem 1, in the troubleshooting Section.

1. Retrieve brain block of interest, stored at room temperature (appr. 23°) submerged in paraformaldehyde (PFA) 4% solution.
2. Remove the specimen from PFA solution and gently dry with a paper towel.
3. Immersed the specimen in perfluoropolyether (Fomblin; Solvay Solexis, Bollate, Italy), in a cylindrical glass container of diameter 25 mm and length 100 mm, allowing for fitting in the vendor’s receiver coil.
4. To avoid strong susceptibility gradients around the bottom of the tube, place Polymethyl-Methacrylate (PMMA) -cylinder (diameter 22 mm, height 15 mm) onto the tube floor to lift the sample into the region with less susceptibility variation. PMMA is used in this context due to its tissue compatible magnetic susceptibility properties.^5^
5. Non-magnetic, PMMA-spheres with 6.4 mm diameter and PMMA-plates of different sizes (thickness 2 mm and 3 mm, width 15 mm, height 3.5 cm and 4. cm) are used to further stabilize the specimen in the tube. Position those longitudinally for ensuring that the sample remains in the center (Figure 1C).
6. Gently move the sample to force out any cavitated air bubbles away from the field of view. After this step, plug PMMA-cylinders in the tube. PMMA-cylinders in this case should be 22 mm in diameter and 15 mm in height to fit the tube diameter.
7. Place the tube with the perfluoropolyether-immersed, secured specimen into the 25 mm bird-cage receiver RF coil of the vendor (Figure 1D) and subsequently mount it into the spectrometer (Figure 1E).

**CRITICAL:** Air bubbles shall be eliminated from the specimen to ensure that the MRI acquisitions quality is not compromised by air-tissue interface susceptibility artifacts. For control, a -survey-scan should be performed to ensure there are no visible bubbles formed around the sample before the full-scanning session (see next step).

**CRITICAL:** Precautions and Handling Instructions - PFA can release formaldehyde vapors that irritate the eyes, skin, and respiratory tract and are recognized as potentially carcinogenic. Preparation and use should always take place in a certified fume hood, with standard protective gear such as a lab coat, chemical-resistant gloves, and safety goggles. Perfluoropolyether is considered chemically inert and of low acute toxicity. However, use of gloves is strongly recommended. For both substances, please follow institutional rules for chemical waste disposal. More information can be found for PFA:

https://www.ehs.harvard.edu/sites/default/files/lab_safety_guideline_paraformaldehyde.pdf

https://www.sigmaaldrich.com/CH/en/sds/sial/p6148?userType=undefined

and for Perfluoropolyether:

https://www.solvay.com/sites/g/files/srpend616/files/2018-07/fomblin-pfpe-lubricants-en.pdf.

### Collecting multi-contrast 9.4T MRI data

**Timing: appr. 56 h**

This step provides the details of the acquisitions and other relevant details for obtaining multimodal, high resolution 9.4T MRI data. Specifically, the following refer to **Bruker Avance Neo 400 WB spectrometer**.

8. Register the required details before the scan, according to the vendor’s interface.
9. Separately for each block, perform the procedure for tuning and matching of the RF coils in the NMR probe head to the ^1^H resonance frequency.
10. Localizing and shimming:
  a. Perform a localizer scan to locate the tissue block with respect to the scanner coordinates. Align the scanning volume to the block’s orientation and location.
  b. Acquire a B0 map. Shim currents are calculated based on this B0-map of the whole sample. The calculated shim is kept for all further measurements.

**Figure 2:**
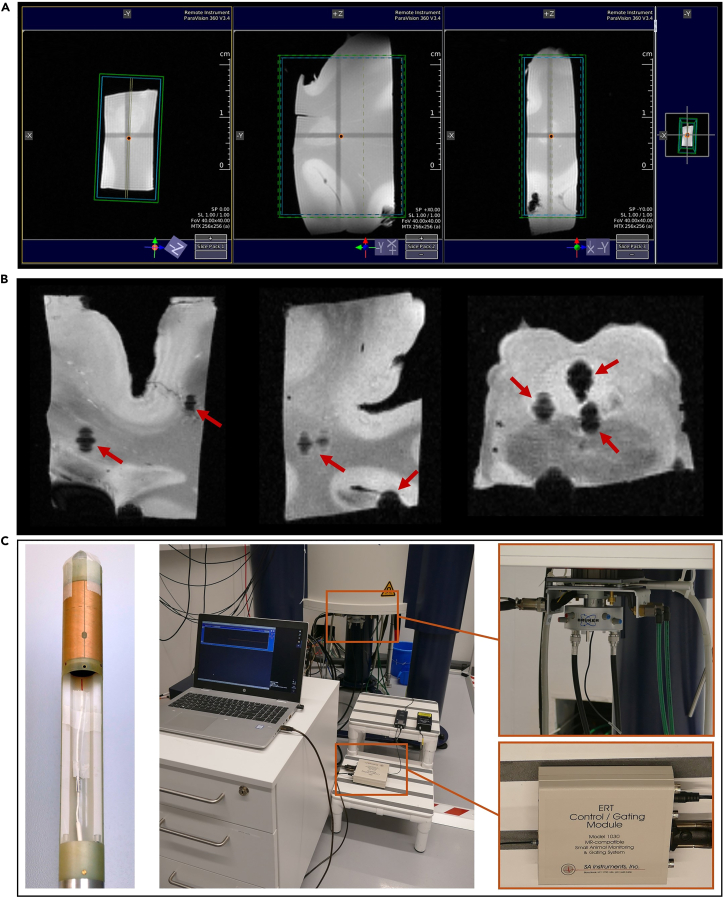
Further details on the MRI measurements setup (A) Indicative shimming volume setting that includes all meaningful regions of the sample. (B) GRE magnitude images presenting examples of persistent air bubbles trapped within tissue compartments, creating sources of artifacts. (C) Temperature monitoring setup and hardware used in our work.

***Note:*** Please note that the shim volume shall include all meaningful regions of the sample (example in Figure 2A).

11. Acquire a 2-echo (2 and 14 ms) **3D Gradient Recalled echo (GRE) localizer sequence** (Acquisition time (TA) = 3 min) is run. These images are inspected in the MRI system console to locate potential remaining bubbles that might obscure the scans quality. If there are no visible air-tissue susceptibility artifacts, the scans can resume. In the event of existing bubbles close or within ROIs, the preparation Step 6 should be repeated to remove it (see Figure 2B for an indicative example).

***Note:*** Only for signal voids that become larger from the 1^st^ to the 2^nd^ echo one can conclude that are air-bubbles or blood clots induced. Alternatively, one can also consult the phase images, for characteristic dipole-like signals of air-tissue interface.

12. Once localization and shimming are done, acquire a **3D EPI-based Diffusion Weighted Imaging (DWI)** protocol (Voxel size = 200μm isotropic, Matrix = 150 × 120 × 56, FOV 30 × 24 × 11.2, TR=1500 ms, TE=24 ms, 10 segments, BW=400 kHz, δ=3.0 ms, Δ=12 ms), with 4 shells:
  a. b=0s/mm^2^ (4 repetitions).
  b. b=2000s/mm^2^ (20 gradient directions).
  c. b=4000s/mm^2^ (30 gradient directions).
  d. b=6000s/mm^2^ (40 gradient directions).

**Figure 3:**
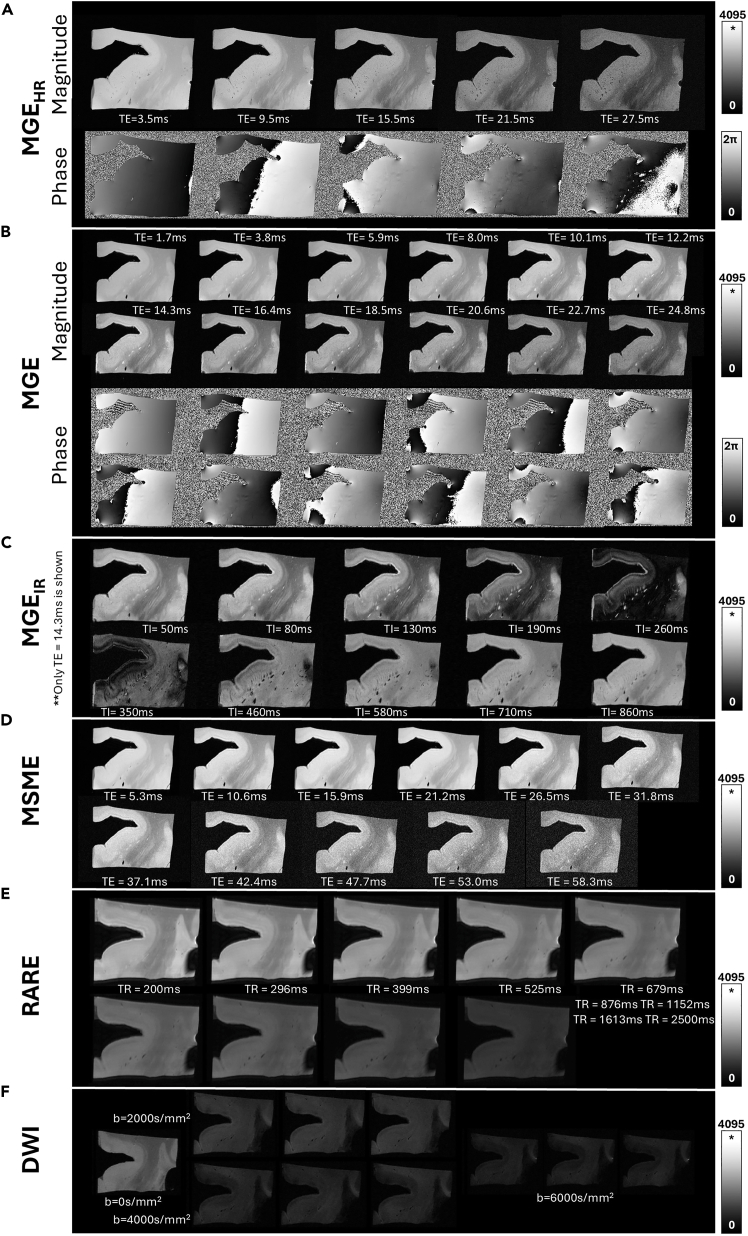
Examples of 9.4T MRI scans of a brain block Exemplary slices of (A) magnitude and phase images of the 5-echo 3D Multi-Gradient-Echo at 70×70×130 μm (MGEHR), (B) magnitude and phase images of the 12-echo 3D Multi-Gradient-Echo at 130×400×130 μm (MGE), (C) magnitude images of Inversion-Recovery 3D MGE (MGEIR) at 130×400×130 μm shown for TE = 14.3 ms at all different inversion times, (D) magnitude images of the 11-echo Multi-Slice Multi-Echo (MSME) at 130×400×130 μm, (E) RApid imaging with Refocused Echoes (RARE) at 130×400×130 μm shown for the different TR values, (F) 3D EPI-based multi-shell Diffusion-Weighted Imaging (DWI) at 200 μm isotropic resolution (one indicative image at b = 0 s/mm^2^ and three for each of the different b-values: 2000, 4000, 6000 s/mm^2^).

TA = 14 h 37 min. Frequency drift correction enabled.

13. Acquire **3D Multi-Gradient-Echo, High Resolution (MGE**_**HR**_**):** Voxel size = 70 × 70 × 130 μm^3^, TR = 320 ms, TE = [3.5, 9.5, 15.5, 21.5, 27.5]ms, α = 35°, 4 averages, BW = 120 kHz, no RF-spoiling, Matrix = 426 × 342 × 86, FOV 30 × 24 × 11.2, TA = 11 h 55 min. Frequency drift correction enabled.
14. Acquire **3D Multi-Gradient-Echo (MGE):** Voxel size = 130 × 130 × 400 μm^3^, TR = 1500 ms, α = 30°, TE = [1.7, 3.8, 5.9, 8.0, 10.1, 12.2, 14.3, 16.4, 18.5, 20.6, 22.7, 24.8]ms, BW = 151 kHz, RF spoiling, Matrix = 230 × 178 × 28, FOV = 30.0 × 23.1 × 11.2 mm^3^, TA = 2 h 4 min. Frequency drift correction enabled.
15. Acquire **Inversion-Recovery-3D MGE (MGE**_**IR**_**):** Voxel size = 130 × 130 × 400 μm^3^, TR = 1500 ms, α = 90°, TE = [1.7, 3.8,5.9, 8.0, 10.1, 12.2, 14.3, 16.4, 18.5, 20.6, 22.7, 24.8]ms, Inversion Time (TI) = [0, 50, 80, 130, 190, 260, 350, 460, 580, 710, 860]ms, BW = 151 kHz, RF spoiling, Matrix = 230 × 178 × 28, FOV = 30.0 × 23.1 × 11.2 mm^3^, TA = 2 h 4 min per TI. Frequency drift correction enabled.
16. Acquire **Multi-Slice Multi-echo (MSME):** Voxel size = 130 × 130 × 400 μm^3^, TR = 1500 ms, BW = 151 kHz, echo spacing = 5.3 ms, Duration 2 h 4 min, Matrix = 230 × 178 × 28, FOV = 30.0 × 23.1 × 11.2 mm^3^, TE = [5.3, 10.6, 15.9, 21.2, 26.5, 31.8, 37.1, 42.4, 47.7, 53.0, 58.3]ms, TA = 2 h 4 min. Frequency drift correction enabled.
17. Acquire **RApid imaging with Refocused Echoes (RARE):** Voxel size = 130 × 130 × 400 μm^3^, RARE 5, TE=12 ms, echo spacing = 6 ms, BW = 151 kHz, TR = [200, 296, 399, 525, 679, 876, 1152, 1613, 2500]ms, Matrix size = 230 × 178 × 28, FOV = 30.0 × 23.1 × 11.2 mm^3^, Matrix = 230 × 145 × 28, TA = 1 h 51 min.
18. Repeat *Step 11* for inspection for artifacts, e.g., formation of bubbles during scan and validation of the sample location compared to the initial scan.
19. Export all data acquired in DICOM format. Exemplary raw data for each aforementioned sequence are shown in Figure 3, for one of the scanned brain blocks.

**CRITICAL:** Temperature is a crucial factor affecting MRI contrast.^6^^,^^7^^,^^8^ If possible, temperature shall be monitored throughout the scan to ensure that there are no extreme fluctuations (<±5°C). If the specimens were stored in a different location or in a refrigerator (4°C–5°C), they should be left in the scanner room for enough time to uniformly acquire the ambient temperature (within the tube with the perfluoropolyether). In case that constant temperature monitoring is not possible for all scans, the temperature before the scan shall be documented and kept stable between different tissue blocks. In our experiments, we started with room temperature and monitored the temperature fluctuations during the scans for 2 brain blocks with specialized probes, to ensure that our MRI protocol does not dispose excessive heat in the tissue (see images of the setup in Figure 2C). In our case, the temperature was constant, around 23°C.

**CRITICAL:** Throughout the measurement, the user *must* ensure that the specimen entirely remains within the FOV, to ensure managing the unlikely cases of unwanted sample displacement -due to potential vibrations during scan, displacement of the PMMA holders allowing the sample to rotate or subjected to gravity, migration of air bubbles, etc.- and uncorrected frequency drifts.

**CRITICAL:** Visual checking for each contrast acquired is *strongly recommended*, to avoid continuation of the scans in case of newly formed bubbles, tissue misplacement, or other issues that can arise. In the case of formation of new significant bubbles close to the sample, it is recommended to stop the measurement, proceed on removing the bubble by gently tilting and shaking of the sample and repeat the acquisition of the current contrast from the start.

***Optional:****Restructuring of the MRI DICOMs*.

The user is provided with a folder containing MATLAB scripts (can be downloaded from: https://github.com/LukasSchoenenberger/IHC-Analysis-Pipeline/tree/main/Optional-Step__DICOM-Data-Restructuring-Script) that allows for derivation of a structured filesystem, with all DICOMs transformed to *NIfTI* format, for easier handling in the eventual steps of registration with histology-derived semi-quantitative maps and further image processing. Running the script ‘*DICOM_data_restructuring_MRI.m’ in* MATLAB (after replacing the input main directory within the script):

 1. Emunerates all DICOM sub-directories in the input main directory.

 2. Runs the command ‘dcm2niix’ in each directory, which converts the DICOM data directly to *NIfTI* (providing also a.json file with the basic parameters and metadata of the acquisition).

 3. Renames the .nii and .json file with a name which is self-explanatory as to the details of the individual contrast, based on the extracted sequence name and Inversion Recovery/Echo time details found in the .json file.

 4. Moves the DICOM files to a subfolder within the sub-directory.

 5. Renames the subfolders based on the nifty acquired filenames.

However, the user can choose individually whether a manual handling of the image raw data is preferable. This MATLAB script is only tested on raw data exported from *Bruker Avance Neo 400 WB spectrometer*. Note that for the next steps of combining MRI with histology, *NIfTI* files are required.

### Immunohistochemistry and microscopy imaging

**Timing: appr. 24 h**

The full details of the immunohistochemistry processes do not concern the present protocol, the reader should refer to *Galbusera R. et al, 2022*.^4^ However, to ensure the reproducibility of the software presented in the following sections we explain in brief the details of the process below:

After the MRI scan, the lesion-containing blocks are embedded in paraffin and subsequently dissected in slices of 4μm. The basic stains include: Myelin Basic Protein (anti-MBP; Agilent (Dako), Glostrup, Denmark, for myelin (1:100), RRID: AB_2650566), anti-CR3/43 (Agilent (Dako), human HLA-DP, clone CR3/43 for MCHII expressing microglia/macrophages (1:50), RRID: AB_2313661). After incubation with the primary antibody (applied at the dilutions indicated and incubated overnight at 4°C), antibody binding was visualized using biotinylated secondary antibodies, peroxidase-conjugated avidin (Dianova (Vector Laboratories), Goat anti-rat (1:200), RRID: AB_2336812) and 3,3′-Diaminobenzidine (DAB) (Sigma-Aldrich). Double-labelling immunohistochemistry was performed combining DAB (Dako) and Fast Blue (Sigma Aldrich) using an alkaline phosphatase-conjugated secondary antibody (Jackson ImmunoResearch AffiniPure Goat anti-mouse IgG (1:100), RRID: AB_2338447). Hematoxylin was used as nuclear counterstain. Imaging was done using an Olympus BX 41 microscope at 20× objective magnification (Olympus/Evident Slideview VS200). The whole slide digital images were then uploaded to an open microscopy OMERO server (version 5.6.3) for distributed collaboration and morphometry.

### Derivation of semi-quantitative maps from histological stains: Preprocessing

**Timing: ca. 8 h**

This step allows the user to derive semi-quantitative myelin, microglia, and cell density maps from immunohistochemical (IHC) images. The pipeline is tailored for processing IHC images acquired with bright-field microscopy and managed using the OMERO data management system. The IHC processing framework provides certain flexibility for handling input data from other sources. We will briefly discuss alternatives at each processing step. *The custom software has been developed and tested in Ubuntu 24.04.2 LTS, Release: 24.04 on a system with 64bit CPU architecture (13th Gen Intel(R) Core(TM) i7-13800H) and 64 GB RAM.*

**Figure 4:**
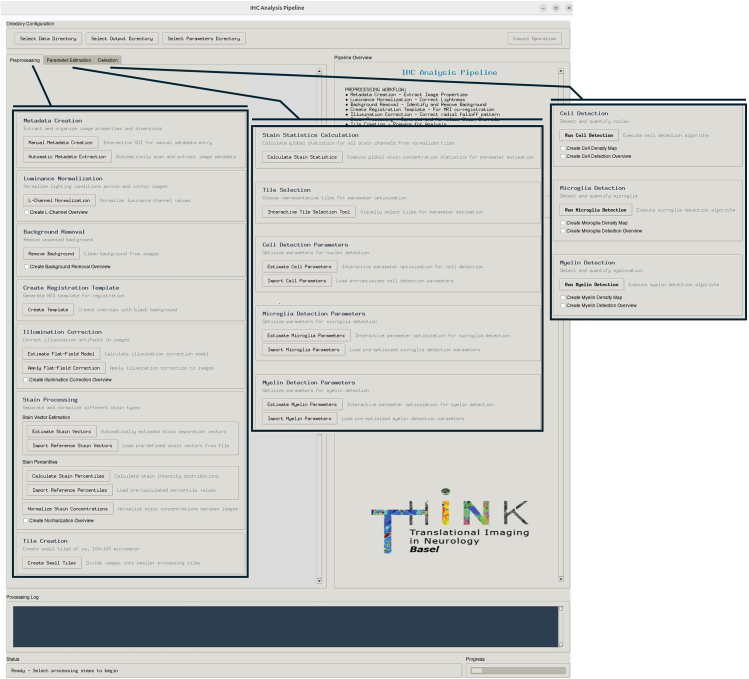
Main Graphical User Interface (GUI) for the processing of histological images (A) The ‘Preprocessing’ tab, (B) the ‘Parameter estimation’ tab and (C) the ‘Detection’ tab.

20. Set-up your working environment.
  a. Download all necessary resources from GitHub.
    i. Navigate to the following URL: https://github.com/LukasSchoenenberger/IHC-Analysis-Pipeline/tree/main.
    ii. Download the repository as a ZIP file. The *requirements.txt* file contains all needed Python packages for the following software.
    iii. Unzip the IHC-Analysis-Pipeline-main.zip.
  b. Set up your work directory.
    i. Copy and place the contents from IHC-Analysis-Pipeline-main/IHC-Analysis-Pipeline-Scripts in your work directory.
  c. Set-up a Conda environment.
    i. Navigate in the terminal console to your working directory.
    ii. Create the Conda environment with:
      >conda env create -f ihc-processing.yml
    iii. Activate the Conda environment with:
      >conda activate ihc-processing
  d. Launch the registration tool:
    i. Navigate in your terminal to your working directory.
    ii. Run the terminal command:
      >python MASTER-SCRIPT.py.
      This will open the main graphical user interface (GUI) of the IHC Analysis Pipeline tool, shown in Figure 4.
21. **Create a file containing all relevant metadata that the pipeline requires in the subsequent processing steps.** There are 2 selections: i) Automatic Metadata Creation and ii) Manual Metadata Creation. The latter allows for entering the metadata input files manually, in the cases that the “Original_Metadata.txt” file from OMERO is not available or another source of input data is used.
  a. Automatic Metadata Creation.
    i. Download the metadata file from OMERO-web. This can be done under *Acquisition/Original Metadata/Download.*
    ii. Save the Original_Metadata.txt file in the “Parameters” folder in your work directory.
    iii. Click on the *Automatic Metadata Extraction* button in the main GUI. This will create a “Metadata.csv” file containing all relevant information needed for further processing steps and save it to the “Parameters” folder. Additionally, this will also create a “Tile-Export-QuPath.groovy” script tailored to this IHC image that can be used to download the whole slide image (WSI) in the correct tile size needed for correction of the illumination artifact.
  b. Manual Metadata Creation.
    i. Click on the button *Manual Metadata Creation* in the main GUI.
    ii. Enter all required metadata manually into the blanks.
    iii. Click on the *Save Metadata* button. This will generate the “Metadata.csv” and the “Tile-Export-QuPath.groovy” script and save them to the “Parameters” folder.

**CRITICAL:** Do not change the name of the “Original_Metadata.txt” file as the script will search for this file name in the automatic metadata extraction step.

**CRITICAL:** The “Tile-Export-QuPath.groovy” script is automatically designed to download tiles that correspond to the original acquisition tile size of the microscope. The exact tile size is crucial for further correction steps. If the user obtained input data from other sources, he or she must make sure that the tile size corresponds to the original acquisition tile size of the microscope. If this is not available, the illumination artifact correction (Step 21) is not applicable and shall be skipped.

22. Download your input data.
  a. Make sure to have QuPath and QuPath’s OMERO extension installed on your computer. A guide on how to set up the OMERO extension can be found in QuPath’s documentation (https://qupath.readthedocs.io/en/0.4/docs/advanced/omero.html).
  b. Open QuPath and connect to your OMERO server.
  c. Create a QuPath project.
  d. Import the WSI from your OMERO server into your QuPath project.
  e. Drag and drop the “Tile-Export-QuPath.groovy” script into QuPath.
  f. Click on the *Run* button to run the script. The files will be downloaded automatically in the correct size to “Data/Tiles-Medium” in your work directory.
23. Apply the luminance correction to normalize the intensity of your input tiles.
  a. Press the button “*L-Channel-Normalization”* in the main GUI.
  b. This will convert the input tiles from RGB to CIELAB image space, normalize the intensity of the L-channel, using global min-max normalization. After this, the L-channel normalized images are converted back to RGB space.
  c. The new normalized images are saved to the sub-directory “Data/Tiles-Medium-L-Channel-Normalized”.

**CRITICAL:** Enhanced comparability between IHC images (from the same or different blocks, stained with the same methodology) can be achieved by normalizing the intensities with respect to a reference IHC image. For this purpose, the user should determine one reliable IHC image with good staining quality as a reference. Normalizing the L-channel helps in streamlining subsequent processing steps like the optical density (OD) based background removal and the stain-concentration normalization. This specific step can be skipped if the user is applying the analysis to one and only IHC image.

24. Remove the background in your image to avoid disturbances in the stain separation (step 24).
  a. Click on the *Remove Background* button in the main GUI. This will launch the following consecutive steps automatically:
    i. Estimate the RGB values of typical background of the WSI by calculating the channel averages of the first tile (tile_r0_c0.tif).
    ii. Sampling 10% of the pixels per tile and estimate a global OD histogram based on the sampled pixels.
    iii. Determine the largest frequency drop in the histogram to estimate a threshold for background separation.
    iv. Use the estimated OD threshold to create a background mask and set all background pixels to value 255 in the RGB space.
    v. The background removed tiles are saved to “Data/Tiles-Medium-L-Channel-Normalized-BG-Removed”.

**CRITICAL:** We assume that the first downloaded tile of the WSI (tile_r0_c0.tif) does not contain tissue, since this is very common in digital histology, and it was always the case in our acquisitions. If this is not the case in your acquisition, you should manually adjust the “analyze_rgb_background” function in the “Background-Removal.py” script to use a representative background tile.

**CRITICAL:** The OD based background removal relies on the assumption that white appearing background pixels with low OD values form a high frequency cluster in the histogram compared to actual tissue pixels. Depending on the input data, the user might need to adjust the number of histogram bins for a reliable background threshold estimation. We strongly recommend checking the “OD-Histogram.png” and the “Registration-Template.jpg” to validate the background removal quality before proceeding, as the correction of the illumination artifact arising from radial falloff of the microscope light source requires adequate background removal. Background removal with too high OD threshold value and consecutive loss of weakly stained pixels can lead to poor illumination artifact correction (in Step 21). See also problem 2, in the troubleshooting Section.

***Note:*** The background is not actually ‘removed’ but rather normalized to a uniform value that allows for its systematic identification and exclusion in the subsequent processing steps.

25. Create a WSI template for registration with the MRI.
  a. Select the *Create Template* button in the main GUI.
  b. This will create a down-sampled overview of the background removed and L-channel normalized tiles, where background pixels are set to zero values.

***Note:*** In principle any WSI overview can be used for registration purposes. We recommend using this template, since black background provides clearer tissue contours after NIfTI conversion and hence simplify the registration task.

26. Correct the illumination artifact arising from radial falloff of the microscopy light source.
  a. Select the *Estimate Flat-Field Model* button in the main GUI. This will automatically estimate a flat field distortion model that can be used to correct the shading artifact.
  b. Select the *Apply Flat-Field Correction* button in the main GUI.
  c. The illumination artifact corrected tiles are saved to “Data/Tiles-Medium-L-Channel-Normalized-BG-removed-Illumination-Corrected”.

**Figure 5:**
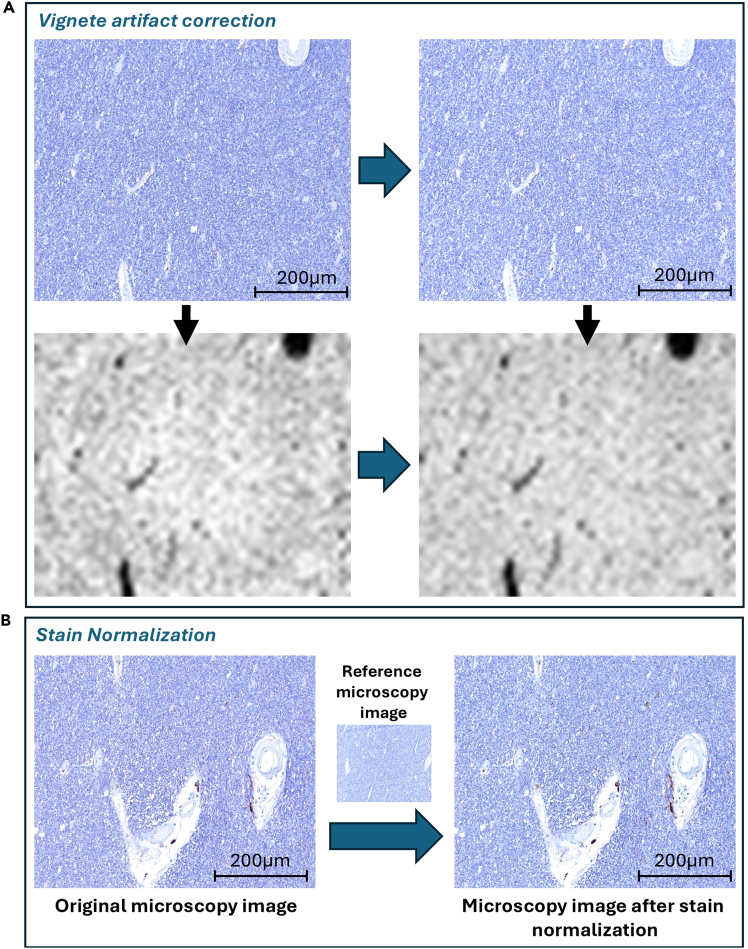
Histological image preprocessing, correction, and normalization (A) The illumination artifact in digital slide images is a result of the intrinsic uneven illumination of the acquisition system, characterized by a radial reduction in brightness from the center toward the edges. In the first row, the uncorrected image and the derived myelin density map of the region are presented. The map indicates a prominent radial brightness reduction. The map based on the corrected image in column 2 shows no sign of illumination inhomogeneity. (B) A before-and-after example of stain normalization of a random ROI from an IHC image acquired during our experiment (optional step to enhance comparability in case of processing histological images of more than one brain samples).

***Note:*** The illumination artifact in digital slide images (an example is depicted in Figure 5A) arises from the intrinsic uneven illumination of the acquisition system due to the radial reduction of brightness from the center point towards the edges (sometimes also referred to as vignette artifact^9^). This artifact becomes more evident when scans acquired from different FOV across the sample are stitched together, thus creating a grid-like pattern known as mosaicking artifact.^10^ Without correction, this artifact causes unreliable intensity measures across the WSI impeding any quantification attempt.^11^ We adapted the method for shading correction detailed in *Tak et al. 2020*.^12^ We excluded in our implementation background pixels with value 255 (since these correspond to our removed background) and we added a constraint in the model selection process to remove candidates with no variation in the intensity profile of each channel (artificially uniform models).

**Figure 6:**
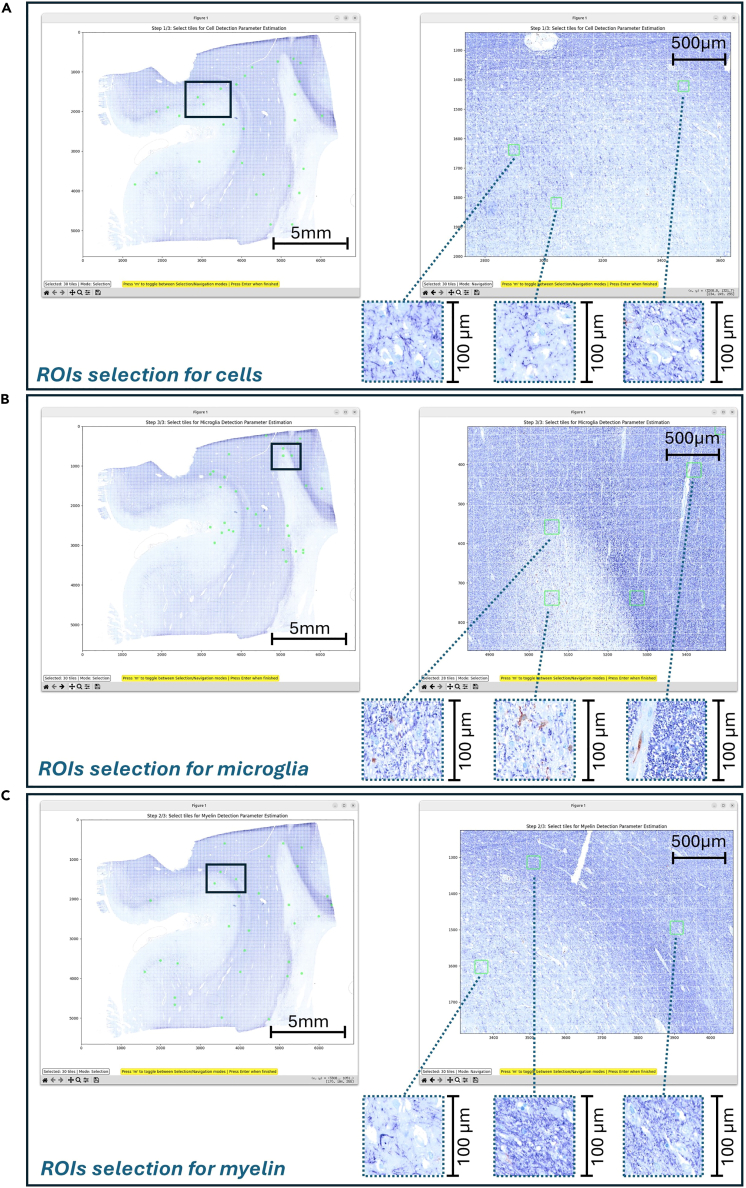
Parameters estimation for informing the algorithmic detection of cells, myelin and microglia Image depicting the interface and exemplary selection of 100 × 100 μm tiles for parameters adjustment, for detection of (A) cells, (B) microglia and (C) myelin. Note that the shown tiles indicate the heterogeneity that the selection must involve, for better parameters estimation used in the next steps of the processing.

**Figure 7:**
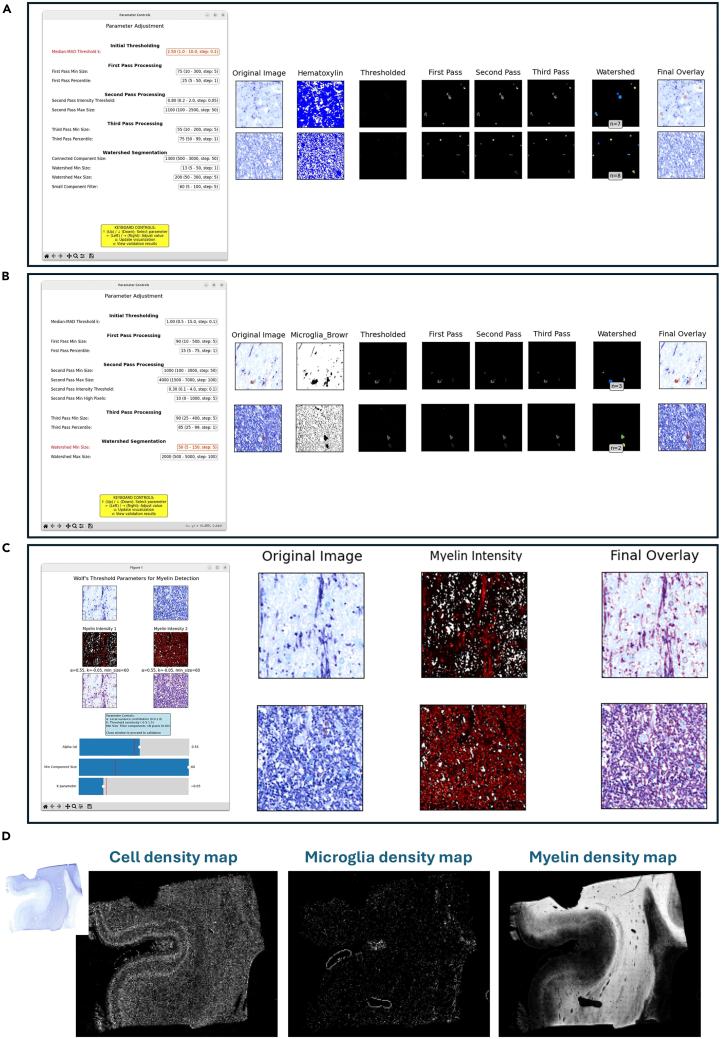
GUI and algorithmic steps for detection of cells, myelin, and microglia Image depicting the user interface and the separate steps for detection of (A) Cells, (B) Microglia, and (C) Myelin, in two exemplary 100×100 μm tiles for each. The selected heterogeneous regions indicate good performance of detection in different cases. (D) The final derived maps for cell density, microglia, and myelin are shown for a full brain block histological slice.

Methods Video S1. Tile selection process: Video showcasing exemplary tile selection process on a lesion-containing histological tile, for Step ‘Derivation of semi-quantitative maps from histological stains: Parameter Estimation’, Sub-step 1

Please note that this step is time-consuming for most systems.

27. Estimate the stain vectors used for separating the stain channels.
  a. Import reference stain vectors by clicking on the *Import Reference Stain Vectors* button in the main GUI.
    i. You can import a “*reference_stain_vectors.txt*” file by using the file browser.
      ***Note:*** This quick method is designed for using stain vectors from your reference WSI that already matches the format of the pipeline.
    ii. You can manually enter the reference stain vectors in a GUI if you not already generated a “reference_stain_vectors.txt” file in standard format.
      ***Note:*** Confirming the input will automatically generate a “reference_stain_vectors.txt” file in the correct format for the pipeline.
  b. Estimate stain vectors using a Bayesian K-SVD hybrid algorithm by clicking on the *Estimate stain vectors* button in the main GUI.
    i. After the algorithm found a stain separation, the user must label the identified stain vectors correctly in a corresponding GUI.
      ***Note:*** An overview of a stain separation example and the results of the convergence analysis are saved to “Results/EBKSVD” for validation.
      ***Note:*** The colorimetric analysis used in this pipeline for the quantification of microstructural tissue components builds on color deconvolution-based stain separation. Color deconvolution is based on the monochromatic Beer-Lambert law that relates the absorbance (or OD) of a material to its concentration**.**^13^ For an IHC image, and according to the Beer-Lambert law, the following equation describes the intensity for each pixel (***Y***):
      Y=CM,
      where ***M*** is the $l_{2}$ normalized optical density matrix of the stains where each row represents a specific stain and each column the optical density as detected by the red, green, and blue channel for each stain. ***C*** denotes the stain concentration matrix where the *n*-th column of ***C*** represents the stain contribution of each stain to the *n*-th pixel intensity in the image. The color deconvolution matrix ***D*** is defined as the inverse of ***M*** and can be used to solve for the stain concentration of a specific pixel:
      C=DY.
      In this pipeline we implemented an approach from *Pérez-Bueno et al. 2022*^14^ for blind color deconvolution (BCD) using a Bayesian K-SVD (BKSVD) algorithm. We adapted the method to work with the deconvolution of three stains instead of two and we replaced the fixed OD threshold for background removal with a histogram-derived OD threshold described in the background removal section. Additionally, the pipeline uses a data-driven initialization of the variational K-SVD algorithm and the empirical K-SVD algorithm is employed in later iterations to refine ambiguous pixels. More information on further techniques for BCD can be found in the review from *Hoque Z. et al. 2024*.^15^
      ***Note:*** The reference stain vectors are required to initialize the color deconvolution pipeline and influence the subsequent stain separation. When analyzing different IHC images stained with the same chromogen, we recommend defining one reference IHC image and using its stain vectors as the reference stain vectors for all the other IHC images processing. To obtain an initial guess of the stain matrix for the reference IHC image, the user can, for example, use standard stain vectors from literature or use one of the many other methods described for color deconvolution. We used QuPath’s implementation of the landmark approach from *Ruifork and Johnston, 2001*^16^ for estimating an initial approximation for the stain vectors. The user can alternatively initialize the algorithm using non-negative matrix factorization (nmf) by running the EBKSVD script standalone and specifying the “--init-method” argument to “nmf.
      **CRITICAL:** We strongly recommend checking the stain separation results and the convergence analysis plots before proceeding with the subsequent processing steps.
28. Calculate relevant stain intensity percentiles in the separated stain channels.
  a. Import reference stain percentiles by selecting “*Import Reference Stain Percentiles”* in the main GUI.
    i. You can import a reference stain percentile file by using the file browser.
      ***Note:*** This quick method is designed for using stain percentiles from your reference WSI that already matches the format of the pipeline.
    ii. You can manually enter the reference stain percentiles in a GUI if you not already generated a reference stain percentile file in standard format.
      ***Note:*** Confirming the input will automatically generate a *reference_stain_percentile.json* file in the standard format of the pipeline.
  b. Calculate stain percentiles of the given WSI by sampling 10% of the pixels from each tile.
    i. Click the *Calculate Stain Percentiles* button in the main GUI.
      ***Note:*** Stain percentiles are required for the stain normalization step that normalizes stain concentrations between different IHC images, which is crucial for enhancing comparability between experiments. This step is not necessary if a single IHC image is analyzed.
29. Normalize the stain concentrations with respect to a reference IHC image.
  a. Click on the *Normalize Stain Concentration button* in the main GUI.

***Note:*** The stain concentration normalized tiles are saved to “Data/Tiles-Medium-L-Channel-Normalized-BG-removed-Illumination-Corrected-Stain-Normalized.”

***Note:*** To enhance comparability between different IHC slices, the pipeline offers the possibility to normalize stain concentrations using a similar approach as described in *Pérez-Bueno et al. 2022*.^14^ Instead of using the 99^th^ percentile, we use the 99.9^th^ percentile as our empirical analysis showed that this percentile better capture the significant range of stain intensity. A before and after example of stain normalization is shown in Figure 5B on a random ROI from an IHC image acquired within our experiment.

30. Dividing the input image tiles into smaller tiles of approximately 100 × 100 μm (360 × 360 pixels).
  a. Click on the *Create Small Tiles* button in the main GUI.

***Note:*** This will automatically split up the input tiles and rename them correctly based on the available metadata file.

***Note:*** The selected tile size for consequent analysis and derivation of the density maps matches *roughly* the achieved MRI resolution. The user may adjust the tile size according to his/her requirements by running the “*Create-Small-Tiles.py*” script as a standalone script with the according parser argument or by adjusting the parser arguments default value manually in the “main” function and run it via the “*Master-Script.py*” script.

### Derivation of semi-quantitative maps from histological stains: Parameter estimation

31. Select representative small tiles for adjusting the parameters of the detection steps 30 to 32 and calculate the global stain statistics of the normalized IHC image.
  a. Click on the *Interactive Tile Selection Tool* button in the main GUI to create a down-sampled overview of the WSI with a grid indicating the boundaries of the small tiles.
  b. The user can toggle between selection mode and navigation mode by pressing the “m” key on the keyboard.
  c. Select tiles for the parameter adjustment by clicking on the tiles you want to use.
  d. After completing your selection press enter to confirm.
    ***Note:*** The selected tiles will be saved together with a cached version of the overview for faster re-creation.
  e. Select the *Calculate Stain Statistics* button in the main GUI.
    ***Note:*** The statistics file is automatically saved to the “Parameters” folder. The statistics are subsequently used for improved visualization of the stain channels in the parameter estimation steps.
    **CRITICAL:** The parameter adjustment is performed on a set of representative image tiles. It is strongly recommended to select at least 30 tiles for each detection algorithm as the adjustment and validation of the parameters is iterative. The selected tiles should capture as much heterogeneity of the IHC image as possible, since the detection parameters should be generalizable to the entire image (or at least all ROIs with different staining characteristics). We recommend sampling different tissue regions (including borders) and stain intensities. Figure 6 presents the interface and an exemplary selection of tiles for parameter adjustment and Methods Video S1 showcases exemplary tile selection process on a lesion-containing histological tile.
    ***Note:*** The detection and quantification of structural components is based on the successful separation of the stain vectors, followed by further image processing steps, e.g. thresholding and morphological operations. The parameters for those processing steps require manual adjustment as the ideal parameter combination may differ between different IHC images. Ideally - in the case of processing more than one block - the parameters shall be adjusted once for the reference block and then imported and used on the others (given that the stain concentration has been normalized). The pipeline offers an interactive GUI for the parameter optimization of the different detection algorithms.
32. Determine the parameters for the cell detection step.
  a. Estimate cell detection parameters by clicking on the *Estimate Cell Parameters* button in the main GUI. This will launch an interactive visualization showing the results from each processing step on 5 example tiles (Figure 7A). The user can adjust the parameters in the *Parameter Controls* interface by using the arrow keys on the keyboard. Pressing “u” updates the processing results based on the currently specified parameters. The “v” key launches the validation interface where the user can validate the detection results on a larger set of image tiles. Pressing the “r” key will open the parameter adjustment interface again and allows the user to refine the parameters on a set of different image tiles. Pressing the “m” key will complete the process and save the final selection of parameters to the Parameters folder. The processing steps for cell detection consist of:
    i. Initial Thresholding: Adjust the threshold for the hematoxylin channel with a Median Absolute Deviation (MAD) threshold (k).
    ii. First Pass Processing: Filter out small components below a certain size threshold and remove a predefined percentage of low intensity pixels from the remaining components. A first pass percentile of 10, for example, filters out the 10% lowest intensity pixels within one component.
    iii. Second Pass Processing: Applies an upper threshold for the components mean intensity in the hematoxylin channel and an upper threshold for the component size.
    iv. Third Pass Processing: Calculates intensity-weighted centroids of the components and filters out a pre-set percentage of pixels that are most distant from the centroid. Then a lower size threshold is applied to the components.
    v. Watershed Segmentation: Applies a watershed operation to the components to separate adjacent cells. Then applies a lower size threshold on the watershed separated components to correct any segmentation errors (i.e., unrealistically small components).
  b. Import cell detection parameters by clicking on the *Import Cell Parameters* button in the main GUI.
    i. Navigate to the “*cell_detection_parameters.json*” file of your reference IHC image in the file browser.
    ii. Select the file and confirm the import. This will save the file to the “Parameters” folder.
      ***Note:*** The parameter adjustment should be performed at least once to obtain reasonable parameters on your reference IHC image. For further IHC images within your experimental setup, you can import the parameters from the reference IHC image and use them for processing if the stain concentrations were normalized in advance. We strongly recommend validating the performance of the parameters for each block separately by creating an overview of the detection step (see the optional step ‘*Create overviews for validation’*). This also applies to the microglia parameter adjustment (Step 31) and the myelin parameter adjustment (Step 32).
33. Determine the parameters for the microglia detection step.
  a. Estimate Microglia detection parameters by clicking on the *Estimate Microglia Parameters* button in the main GUI. This will launch an interactive visualization for the parameter adjustment that works in the same way as for the cell detection parameter adjustment described above (Figure 7B). The processing steps for the microglia detection consist of:
    i. Initial Thresholding: Threshold the microglia channel with a median-MAD threshold. The user can adjust the MAD threshold (k).
    ii. First Pass Processing: Filter out small components below a certain size threshold and remove a set percentage of low intensity pixels from the remaining components.
    iii. Second Pass Processing: Filter components that do not satisfy either size requirements or intensity requirements.
      ***Note:*** The size requirement is defined by a minimum size threshold that should be set at a high value to keep only very large components. The intensity requirement is defined by a two-step process: A minimum intensity threshold and a threshold for the number of pixels within the component that must pass this intensity threshold. All components that pass either the size or the intensity requirement are subject to an upper size threshold to remove potential artifacts.
    iv. Third Pass Processing: Calculates intensity-weighted centroids of the components and filters out a pre-set percentage of pixels that are most distant from the centroid. Then a lower size threshold is applied to the components.
    v. Watershed Segmentation: Applies a watershed operation to the components to separate adjacent microglia cells.
  b. Import microglia detection parameters by clicking on the *Import Microglia Parameters* button in the main GUI.
    i. Navigate to the “*microglia_detection_parameters.json*” file of your reference block in the file browser.
    ii. Select the file and confirm the import. The file will be saved to the “Parameters” folder.
34. Determine the parameters for the myelin detection step.
  a. Estimate the myelin detection parameters by clicking on the *Estimate Myelin Parameters* button in the main GUI. This will launch an interactive visualization for the parameter adjustment that works in the same way as for the cell detection parameter adjustment (Figure 7C). There is only one processing step for the myelin detection:
    i. Apply Wolf’s adaptive thresholding to the non-negative pixels in the Myelin channel.^17^ The user can adjust the parameters alpha (local variance weight) and k (threshold sensitivity).
  b. Import myelin detection parameters by clicking on the *Import Microglia Parameters* button in the main GUI.
    i. Navigate to the “*myelin_detection_parameters.json*” file of your reference block in the file browser.
    ii. Select the file and confirm the import. The file will be saved to the “Parameters” folder.

***Note:*** The window size of Wolf’s adaptive threshold for calculating the local statistics is set to 15 pixels by default, since it proved to produce robust results with our pipeline. The user can manually adjust this parameter in the “__init__” function of the “*Myelin-Parameter-Estimation.py*” script if necessary.

***Note:*** All parameter adjustment scripts work with slightly modified stain channels. Intensity values of the stain channels are truncated below 0 and above 3 to exclude negative intensity pixels and very high intensity pixels, respectively, since they don’t contribute to the range of meaningful stain intensities for the detection of biological structures. These values are derived empirically and can be adjusted manually by the user if necessary. The “*reference_stain_percentiles.json*” file may serve as an initial reference for finding a meaningful range of stain intensities for the underlying data.

### Derivation of semi-quantitative maps from histological stains: Detection

35. Apply the detection algorithms to the entire IHC image.
  a. Run the cell detection by clicking on the *Run Cell Detection* button in the main GUI.
  b. Run the microglia detection by clicking on the *Run Microglia Detection* button in the main GUI.
  c. Run the myelin detection by clicking on the *Run Myelin Detection* button in the main GUI.
36. Calculate density maps of the target structures based on the detection results.
  By ticking the *Create Density Map* check box before running the detection step, a density map of the corresponding histology measure will be automatically generated.
  a. Cell density map: The pipeline outputs two versions of the density map: one where the pixel values correspond to the actual cell count per tile and one where this value is scaled to [0,255] for better visualization.
  b. Microglia density map: The pipeline outputs two versions of the density map: one where the pixel values correspond to the actual microglia count per tile and one where this value is scaled to [0–255] for better visualization.
  c. Myelin density map: The pipeline outputs only one density map where the pixel intensity corresponds to the number of myelin positive pixels per tile scaled to [0,255].
    ***Note:*** In the density map each 36 × 36 pixel square correspond to one processed small input tile. Each of those 36 × 36 pixels have the same value, corresponding to the actual count of the histology measure within the respective tile.
    ***Note:*** We strongly recommend the derivation of density maps for further analysis. This approach scales the histology measurements to a range that is comparable with the MRI resolution while maintaining the region-specific microstructural characteristics of the tissue. Examples of the density maps are shown in Figure 7D.
    ***Optional:*** Create overviews for validation.
    The user can create down-sampled overview images of the WSI after each processing steps. These overview images can serve as a validation and are highly recommended, especially for the background removal, stain concentration normalization and the final global detection steps. To do so, the user can just tick the checkbox *Create Overview* in the main GUI before running the corresponding processing step.

### Registration of histology-derived semi-quantitative maps, histological images, and histology-derived segmentations with MRI contrasts

**Timing: ca. 45 min**

This step allows for co-registering histology images and derived maps (see previous steps) to the corresponding slice of the MR volume, by identifying the best corresponding slice in the MRI volume, performing a linear registration step for coarse alignment, and refining the registration with a non-linear landmark-based registration step.

37. Set up your working environment:
  a. Download all necessary resources from GitHub.
    i. Navigate to the following URL: https://github.com/LukasSchoenenberger/IHC-Analysis-Pipeline/tree/main
    ii. Download the repository as a ZIP file. The *requirements.txt* file contains all needed Python packages for the following software.
    iii. Unzip the IHC-Analysis-Pipeline-main.zip.
  b. Set up your work directory.
    i. Copy and place the contents from IHC-Analysis-Pipeline-main/IHC-MRI-Registration-Scripts in your work directory.
    ii. Place your input data into your work directory. The input data consist of:
       A reference MRI image (necessary).
       An overview image of the histology slice that is registered (necessary).
       Folders containing further histology derived images intended for registration (optional).
       A folder containing further MRI sequences intended to register to the reference MRI (optional).
  c. Activate the Conda environment:
    i. Navigate in the terminal console to your working directory.
    ii. Create the Conda environment with:
      >conda env create -f ihc-mri-registration.yml
    iii. Activate the Conda environment with:
      >conda activate ihc-mri-registration
      **CRITICAL:** MR images must be in NIfTI format (.nii or .nii.gz) and histology images must be in PNG, JPEG or TIFF format.
      ***Note:*** You can use the “Background-Removal-Overview-Black.jpg” image from the previous step as a source histology image.
38. Launch the registration tool.
  a. Navigate in your terminal to your working directory.
  b. Run the terminal command:

>python MASTER-SCRIPT.py.

**Figure 8:**
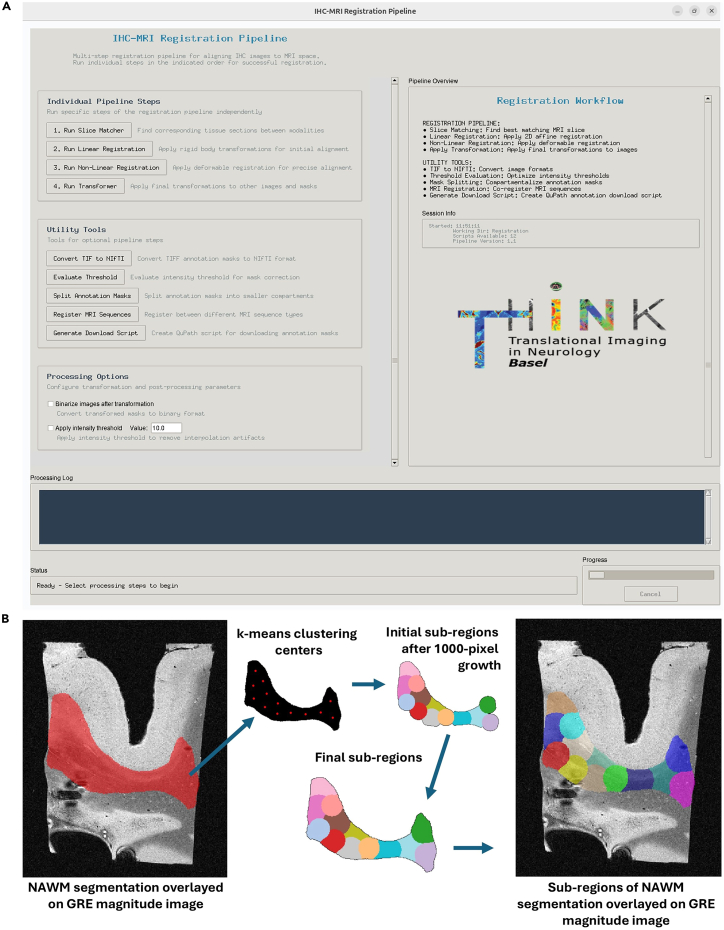
GUI for registration and segmentation masks splitting (A) main GUI for the immunohistochemistry and MRI contrasts co-registration tool. (B) An example of the functionality for splitting the segmentations into equally sized subregions for patch-wise analysis of ROIs. This functionality helps to effectively capture potential heterogeneity of big ROIs without resorting to voxel-wise analysis, avoiding some potential pitfalls of the microscopic (histopathology) to mesoscopic (MRI) analysis assumptions.

This will open the main graphical user interface (GUI) of the registration tool (Figure 8A).

39. Download annotation masks from QuPath.
  a. Annotate your ROIs in QuPath.
  b. Click on the *Download Annotation Masks* button in the main GUI.
    i. Fill in the names of all annotations you want to download from QuPath.
    ii. Confirm your selection. This will automatically generate a “Download-Annotation-Masks.groovy” script in your work directory.
  c. Drag and drop the “Download-Annotation-Masks.groovy” script into your QuPath window and click *Run* in the script editor window.

***Note:*** The annotation masks will be downloaded into the “Annotation_masks” folder in your work directory.

40. Match the IHC image to the best corresponding MRI slice.
  a. Click the button *Run Slice-Finder* in the main GUI.
  b. Select your reference MRI volume in the file browser.
  c. Select your source IHC image in the file browser.
  d. The slice-finder interface will open, and the script will convert the source IHC image to NIfTI format and automatically identify the anatomical plane containing most non-zero voxels, which correspond in case of imaging tissue blocks to the plane of interest.
  e. Select the best corresponding slice in the MRI volume. Therefore, you have the option to:
    i. Select one of four similarity metrics for automated slice identification. By default, *NMI* (normalized mutual information) is selected. To use another similarity metric, select either *NCC* (normalized cross correlation), *SSIM* (structural similarity metric) or *Feature* (using OpenCV’s Oriented FAST and Rotated BRIEF (ORB) algorithm) and click the button *Try another similarity metric* in the slice-finder GUI.
    ii. Select the best matching slice manually by visual inspection.
      ***Note:*** Clicking on the button *Manually select a slice* will open the manual-selection interface where you can select a slice using the sliding bar on the bottom. This process is guided by displaying all four similarity metric values for the current slice.
  f. Confirm your selection by clicking on the *Confirm – Use this slice* button.
  g. Select the correct orientation of the histology image.
    ***Note:*** All possible orientations of the histology image are displayed together with the reference MRI slice. Choose the correct orientation by pressing the corresponding number key on your keyboard or alternatively clicking on the respective bullet point in the option list.
  h. Select the best matching slice again, this time with the correctly oriented source image. The interface is the same as in the first round of slice selection.
  i. Confirm your selection by clicking on the *Confirm – Use this slice* button.
    ***Note:*** This will save a summary image, the NIfTI converted IHC image and the matching info containing information about anatomical plane, orientation, and slice level to the “Match_slice_results” folder for future reference.
    See also problem 3, in the troubleshooting Section.
    ***Note:*** This step reduces the complex 2D-to-3D registration problem, to a 2D-to-2D registration problem. Based on the significant difference in slice thickness between histology and MRI (in our case 4 μm vs. 70 μm, correspondingly), we assume that a single histology slice is not likely to span across more than a single MRI slice.
    ***Note:*** The script automatically applies several preprocessing steps to the histology image to improve the registration process. These preprocessing steps consist of grayscale conversion, intensity normalization, histogram equalization and edge-enhancement.
    ***Note:*** In case the orientation selection proves very difficult by visual inspection, the processing log displays the Histogram of Oriented Gradients (HOG) values for each orientation after finishing the process, indicating the most similar orientation as a helpful guidance in the decision process.
41. Perform an initial linear registration step for coarse alignment.
  a. Click the button *Run Linear-Registration* in the main GUI.
  b. Select your reference MRI volume in the file browser.
  c. Choose the best registration result. The linear-registration tool automatically runs linear registration guided by three similarity metrics and an identity transform. Thus, the user can select from four options for the linear registration step:
    i. Normalized mutual information (NMI).
    ii. Normalized cross correlation (NCC).
    iii. Histogram of oriented gradients (HOG).
    iv. Identity transform (None).
  d. Confirm your selection by pressing the *Confirm Selection* button. This will save an overview image, the linearly transformed IHC image and the calculated transformation matrix to the “Linear_registration_results” folder for future reference.

***Note:*** The initial linear registration step not only simplifies the landmark-based registration task, but also helps in avoiding overfitting and generating an unrealistic deformation field.

***Note:*** Each of the algorithms apply a 2D affine registration with 6 degrees of freedom allowing for translation, rotation, scaling, and shearing. The algorithms used are:

 1. SimpleITK’s NMI as similarity metric and gradient descent for optimization.

 2. SimpleITK’s NCC as similarity metric and gradient descent for optimization.

 3. OpenCV’s HOG features as similarity metric and enhanced correlation coefficient (ECC) to align the HOG representations using an iterative Gauss-Newton optimization approach.

 4. Identity transform.

***Note:*** In the linear-registration interface the four columns correspond to the four registration options. The first row shows the transformed histology image and the registration evaluation based on NMI and SSIM. The second row displays an overlay image of the registration result (red) and the reference MRI slice (green). The third row shows a difference heatmap between the transformed histology images and the reference MRI slice.

42. Perform a non-linear registration step for registration refinement.
  a. Click the button *Run Non-linear-registration* in the main GUI.
  b. Select your reference MRI volume in the file browser.
  c. Place landmarks in the non-linear-registration interface.
    i. Place alternating landmarks on corresponding anatomical structures in the histology and in the MRI image. Start with the histology image.
    ii. You can use the mouse wheel to zoom and right-click + drag to pan.
    iii. Misplaced landmark pairs can be removed by pressing the backspace key.
  d. Complete the step by pressing enter or clicking on the *Perform Non-Linear Registration* button.

***Note:*** This will save an overview image, the non-linearly transformed histology image and the calculated transformation matrix as well as the deformation field to the “Non-linear_registration_results” folder for future reference.

***Note:*** The number of landmarks required for good registration results generally depends on the initial alignment of the input images. In our experience good results can be achieved by efficient placement of at least 30 landmarks (preferably >50). Examples of optimal and suboptimal landmark placement can be found in Figure S1. Placing too many landmarks on misaligned input images can lead to overfitting and should be avoided. Furthermore, solving the system of linear equations scales in Ο(n3) and thus gets computationally expensive for too many landmarks.

***Note:*** For the non-linear registration we use Scipy’s implementation of the thin-plate spline transformation to create two radial basis function objects (one for the x- and one for the y-coordinate) and solve for the parameters (affine transformation coefficients and weights of the non-linear part) while promoting smoothness. A deformation field is calculated to match each source pixel to the target space using bilinear interpolation. Any pixels outside the valid interpolation regions (NaN values) are filled using nearest-neighbor interpolation.

43. Apply the transformation to other histology derived images.
  a. Place the images you want to transform into a folder.
  b. Transform your images into correctly placed and oriented NIfTI files.
    i. Click on the *Convert TIF to NIfTI* button under “Utility Tools” in the main GUI.
    ii. Select the input folder in the file browser.
    iii. The output NIfTI files are stored again in the same folder.
  c. Click on the *Run Transformer* button in the main GUI.
  d. Select your input folder in the file browser. The linear and the non-linear transformations will be applied to all NIfTI files in the selected input folder in the correct order.
  e. The output files are saved to the “Transformation_results” folder.

**CRITICAL:** A reasonable size of the subregions for the annotation splitting depends on your reference MRI size and resolution. For our images with in-plane resolution of 130 μm we used subregions of approximately 500 voxels. If necessary, the user can manually adjust the target size by updating the “target_size” parameter in the “grow_region_from_seed” function of the *Segmentation-Splitting.py* script.

***Optional:*** Threshold and binarize annotation masks.

The non-linear registration step can lead to de-binarization of pixel values in binary images due to interpolation effects. To correct for this, the user can threshold the transformed image and binarize the result. Note that the user must first perform a normal transformation (without threshold and binarization) to create a source image for the threshold evaluation. Then evaluate a proper threshold using the *Evaluate Threshold* utility tool. The user can select a transformed mask and the reference MRI volume in the file browser. After selection, an interface is launched where the user can set a satisfying threshold upon visual inspection using the sliding bar at the bottom. After determining an optimal threshold, the user can enter the threshold in the main GUI and tick the checkbox *Apply intensity threshold* as well as the checkbox *Binarize images after transformation*. When clicking the *Run Transformer* button with these two options enabled, the output images are automatically thresholded based on the given intensity threshold value and binarized. The output images are saved to the “Transformation_results” folder.

***Optional:*** Split annotation masks into subregions.

The pipeline offers the possibility to split the annotation masks into equally sized subregions thus supporting patch wise analysis of ROIs (Figure 8B). This step requires properly binarized annotation masks as input, so the optional step “Threshold and binarize annotation masks” should be performed in advance.

To split the annotation masks, click on the *Split Annotation Masks* button in the main GUI. Select the annotation masks you want to split in the file browser. These masks are then divided into subregions of approximately the same size, where each subregion is assigned a distinct voxel value. The algorithm generates potential seed points based on a distance transform of the pixel values of the mask and then uses k-means clustering to select a set of optimal seed points based on the subregion target size. Subregions are then generated by independent concentric growth from all seed points simultaneously. After creation of these initial balanced subregions, any remaining available pixels are assigned to the nearest adjacent subregion using a boundary growing approach to maintain region continuity.

***Optional:*** Co-register other MRI sequences to the reference MRI image.

In this optional step, the user can co-register further MRI sequences with different resolution of the same tissue block to the reference MRI volume. Aligning the voxel dimensions between sequences is necessary for applying the annotation masks properly.

Create a folder in your work directory and place the source MR images you want to register into the folder. Then click on the *Register MRI sequences button* in the main GUI and *s*elect your input folder and your reference MRI volume in the file browser.

The script will automatically resample all input files with the target MRI dimension and resolution. The results are saved to the “MRI_sequences_registered” folder.

***Note:*** This step only resamples the source image by applying an identity transform to match the dimensions and spacing of the reference MRI volume.

## Expected outcomes

After **Steps 1 and 2 (Sub-steps 1–19)**, one should have collected multicontrast anatomical MRI data (appr. 3.2 GB per brain tissue block). The scans acquired may serve as basis for the derivation of a great number of MRI derivative contrasts, like myelin water fraction (MWF), classic and advanced diffusivity metrics and volume fractions, R2∗, R2, R1 relaxation rates, etc., at axial resolution beyond 200 μm. **Step 3** will allow for staining and imaging of slices from the relevant brain tissue blocks.

**Steps 4,5,6 (Sub-steps 20–34)** are explanatory for the usage of a custom, adaptable software, with which the user can derive semi-quantitative density maps of myelin as well as robust maps of microglia and cells count. These maps can then be used in conjunction with MRI-derived contrasts as a robust alternative to the simple stain intensities. After successfully running these steps, one should have produced i) a myelin density map, ii) a microglia count map and iii) a cell count map, at a 100 × 100 μm resolution.

**Step 7 (Sub-steps 35–41)** describes a custom registration tool for registering histological images to the MRI space. The user can selectively register not only the histological slices, with automated solutions on the selection of the MRI volume corresponding slice, but also segmentation masks created directly on the IHC imagery. Additionally, these masks can be further segmented into smaller subregions of the same label, allowing an alternative for capturing the heterogeneity of tissue without resorting to voxel-wise analysis - if not necessary. After successfully running Step 7, one should have produced i) registered myelin density map, ii) registered microglia count map, iii) registered cell count map, iv) registered IHC image and optionally, v) registered segmentation masks, to the best-matching MRI slice, at 70 × 70 μm^2^ axial resolution.

## Limitations

### Postmortem MRI-related limitations

Post-mortem brain tissue exhibits markedly different magnetic resonance (MR) properties compared to *in-vivo* tissue. First, a reduction in diffusivity has been consistently observed in post-mortem diffusion-weighted imaging. In addition to the effect of lower temperature, the physical diffusion process is affected by the chemical alterations of the fixation. Based on both effects, the water diffusion coefficient at ambient temperature is reduced by about 30%–50% of the *in vivo* value at body temperature after *in-situ* perfusion fixation.^18^ Post-mortem interval and the fixation process itself have also been shown to affect diffusivity and diffusion anisotropy in WM.^1^^,^^3^^,^^19^^,^^20^ Second, the fundamental MR relaxation times T1 and T2 are similarly shortened in post-mortem tissue, reflecting changes in tissue hydration, protein cross-linking, and increased magnetic susceptibility differences. These effects can be partially mitigated by phosphate-buffered saline (PBS) soaking of the specimen.^21^ Such an approach has uncontrollable effects on the fixation and diffusivity increase and was not employed in the current methodological protocol as it would constitute another factor of inter-sample incomparability. However, it can provide a solution in cases of measurements on single tissue samples or when the aim is maximizing specific contrasts.^8^^,^^22^

Additional factors such as the method and quality of fixation (e.g., immersion vs. perfusion), storage conditions, and fixation duration can significantly alter tissue contrast and volumetric integrity.^1^^,^^2^^,^^3^^,^^6^ Worth noting is that the actual cause of death and the length of the post-mortem interval may also compromise tissue quality: ischemic injury, inflammation, or autolytic processes potentially affect both MR and histological analyses.^6^

Advanced MR techniques capable of detecting short-T2 components -such as direct imaging of the myelin bilayer^23^ -offer promising specificity for white matter but are not yet employed within our protocols; however, this is considered as a potential future addition. Another addition in the current MRI measurements setup would be an external water compartment that allows for measurement-specific quantification of the water susceptibility which can in turn be used as reference for the derivation of metrics such as Quantitative Susceptibility Mapping (QSM). For post-mortem brain block measurements, using the mean block susceptibility as reference can lead to uncontrolled shifts between diamagnetic and paramagnetic values because of the unbalanced proportions of white and gray matter. The external water reference would help avoid that issue.^24^

An important limitation - not only in our work, but of all tries for bridging MRI and histology, is that MRI captures the bulk nuclear magnetic resonance response of a volume of tissue and not the existence of specific tissue components. Moreover, micro-level analyses such as single-cell identification or mapping of sparse cellular populations are typically disconnected from MR imaging unless those populations form clusters that are spatially resolvable with MRI.

### IHC-related limitations

Mechanical artifacts from tissue sectioning further degrade spatial fidelity in small or delicate brain structures, but also in the macroscopic details of the slice, e.g., splitting. While histology remains the gold standard for cellular-level resolution, it is limited by spatial sampling and does not capture the full microstructural heterogeneity or spatial variability of the lesions.^4^ At the same time, the presence of certain artifacts - at times hard to differentiate from tissue constituents - can limit the value of the histological sample.^25^ High quality histological staining, minimum heterogeneity in staining, maintenance of the microscope/imaging system settings and luminance conditions is required across samples, especially if the aims are towards combining other imaging methods with histopathology.

### Histological image processing-related limitations

Immunohistochemistry (IHC) quantification presents inherent limitations, notably due to the non-stoichiometric nature of chromogen reactions and the influence of light scattering - complicating linearity assumptions in quantification because the Lambert–Beer law holds only for pure absorbers, not scatterers.^26^^,^^27^ In our implementation, the chromogen separation is inherently restricted to a maximum of three stains due to the three-dimensional nature of the RGB color space deconvolution. The detection algorithm used in our analysis depends critically on effective chromogen separability, which in turn relies on adequate stain separation within the RGB image. Therefore, using distinctly colored chromogens is highly recommended, as a greater natural difference in color space improves the robustness and accuracy of the stain separation process. For example, in our dataset, the combination of hematoxylin (HE) as a background stain with the fast blue chromogen for myelin was suboptimal.

Illumination artifacts, particularly vignetting due to radial light falloff, are challenging to correct accurately because the physical size of the artifact cannot be precisely resolved at the given pixel size. Although increasing spatial resolution could improve this approximation, it introduces computational limitations. This may lead to a cumulative offset across rows and columns. However, based on our experience, this level of inaccuracy does not significantly impact the generation of density maps. Additionally, as regards to physical artifacts such as tissue tears, scratches, dust particles, or air bubbles, our pipeline does not currently implement any automated removal methodology. Instead, these artifacts are manually excluded during the segmentation process used to generate region-specific annotation masks.

Registration between histological slices and different image modalities always constitutes a significant computational and methodological challenge.^28^^,^^29^ The 3D nature of histological deformations, local warps and shrinkage are impossible to correct for. However, the pipeline combining linear and non-linear registration we propose, can handle in-plane deformations considerably well, while at the same time the scenario in which histological deformations extend across multiple MRI slices is assumed to be rare due to the substantial disparity in slice thickness and axial resolution between MRI and histology.

Last, in the derivation of density maps, there are specific pitfalls and limitations that should be considered. One notable issue involves interpolation errors that can arise during the co-registration process. Due to the effects of non-linear registration, pixel values in the density maps may slightly alter. As such, the pixel values in the cell and microglia density maps—originally corresponding to actual counts per small tile—should be interpreted with caution. Despite this, cell density maps and microglia density maps can be considered a reliable quantification of the underlying cell distribution as i) interpolation-induced errors are mostly insignificant and cannot lead to major interpretation or quantification changes, ii) they are based on the count of structural components and do not only rely on intensity values of the stain channel. In contrast, the myelin density map should be viewed as a semi-quantitative approximation of the underlying myelin distribution. This is due to longstanding concerns about the linear relationship between staining intensity and the actual concentration of the target protein. Furthermore, multi-stained RGB images do not fulfill the monochromacy assumption required by the Beer-Lambert Law. A thorough discussion of these limitations is beyond the scope of this protocol, but interested readers may refer to relevant discussions in works by *Haub et al. 2015*^30^ or *Landini et al. 2021*.^31^

## Troubleshooting

### Problem 1

• Associated with step: ‘sample preparation for 9.4T MRI acquisitions’, **Sub-step 2.**
• PFA storage can lead to gradual dehydration and excessive brittleness of the brain tissue samples.^32^ Consequently, MRI contrasts are affected by the alterations in water compartments. The effect is especially evident in DWI measurements, where signal-to-noise ratio (SNR) may be reduced.

### Potential solution

Immersing fixed post-mortem brain tissue in phosphate-buffered saline (PBS) may help to rehydrate and equilibrate the tissue, improving image SNR. Unfortunately, no study to date has systematically investigated the effects of PBS immersion time on tissue properties or MRI contrasts. In the literature, PBS washing durations range from several hours up to several days, depending also on the size of the samples (smaller excised blocks or whole brains).

***Note:*** PBS washing will affect the quantitative aspects of the MRI contrasts in an uncontrollable way. This is important to be considered if the aim is the quantitative evaluation of MRI properties between different samples.

### Problem 2

• Associated with step: ‘derivation of semi-quantitative maps from histological stains: preprocessing’, **Sub-step 22.**
• The pre-selected image processing parameters might -at times-lead to inadequate background removal (See Figure S2).

### Potential solution

• The user can adapt the number of histogram bins in the script ‘Background-Removal.py’ (line 256 and line 277):

line 256: hist_values, bin_edges = np.histogram(filtered_values, bins=46)

line 277: plt.hist(filtered_values, bins=46, alpha=0.7, color='steelblue')

• This is a step recommended when the background removal is consistently underperforming over several images within your experiment.
• The user can set a manual background removal threshold by running the background removal as a standalone script with the parser-argument “--manual-threshold”. The latter solution is recommended when the background removal is performing inadequately in single outlier images within your experiment. The relevant command (run on a Linux Bash command line, replace FLOAT by an actual floating point number specifying your desired threshold):

>> python Background-Removal.py --data-dir /PATH TO YOUR WORKING DIRECTORY/Data --output-dir /PATH TO YOUR WORKING DIRECTORY/Results --parameters-dir /PATH TO YOUR WORKING DIRECTORY/Parameters --manual-threshold FLOAT

### Problem 3

• Associated with step: ‘registration of histology-derived semi-quantitative maps, histological images, and histology-derived segmentations with MRI contrasts’, **Sub-step 38.**
• The user should not completely rely on the automatic slice detection. Depending on the underlying tissue structure different similarity metrics may weight different aspects of the image, which can often guide the user’s decision and sometimes provides the ideal match but should never completely replace the holistic evaluation of the user. Additionally, some similarity metrics are influenced by the orientation of the image. In our experience the automatic slice detection provides in ca. 50% of the cases the ideal match.

### Potential solution

In cases of slice mismatch, the user should i) try the usage of different similarity metrics, or, ii) use the manual registration option which is provided in our interface. The latter allows the user to ensure the best match based on visual comparison of the histology and the MRI slices along with an overview of the similarity metrics in real time.

## Resource availability

### Lead contact

Further information and requests for resources should be directed to the lead contact, Prof. Cristina Granziera (cristina.granziera@usb.ch).

### Technical contact

Technical questions on executing this protocol should be directed to and will be answered by the technical contact, Dr. Dimitrios G. Gkotsoulias (dimitrios.gkotsoulias@unibas.ch).

### Materials availability

Not applicable.

### Data and code availability

The MRI data acquired with the present protocol are not openly available. The code described in this protocol associated with the derivation of histological metrics is openly available on GitHub, at https://github.com/LukasSchoenenberger/IHC-Analysis-Pipeline (version of record: DOI: https://doi.org/10.5281/zenodo.17397857).

## Acknowledgments

This work was funded by grants from the Swiss National Foundation (SNF) project: 320030-227901 and Swiss National Foundation (SNF) Excelentia: PP00P3_206151. All collaborators and core facilities that contributed to this work are as follows: Research Center for Clinical Neuroimmunology and Neuroscience Basel (RC2NB), University of Basel, Switzerland; Translational Imaging in Neurology Basel, Department of Biomedical Engineering, Faculty of Medicine, University of Basel, Switzerland; Division of Medical Physics, Department of Diagnostic and Interventional Radiology, University Medical Center Freiburg, Faculty of Medicine, University of Freiburg, Freiburg, Germany; AMIR – Preclinical Imaging Research Center-Core Facility, University Medical Center Freiburg, Freiburg, Germany; and Department of Neuropathology, University Medical Center Göttingen, Göttingen, Germany.

## Author contributions

D.G.G.: conceptualization, methodology, software, investigation, validation, writing – original draft, writing – review and editing, and visualization. L.S.: conceptualization, methodology, software, investigation, validation, writing – original draft, writing – review and editing, and visualization. J.L.: conceptualization, methodology, investigation, validation, and writing – review and editing. I.C.: methodology, validation, and writing – review and editing. E.B.: methodology, resources, and writing – review and editing. D.v.E.: methodology, resources, writing – review and editing, and supervision. V.G.K.: methodology, resources, writing – review and editing, and supervision. M.W.: conceptualization, methodology, writing – review and editing, and supervision. C.S.: resources, writing – review and editing, supervision, project administration, and funding acquisition. C.G.: conceptualization, methodology, resources, writing – review and editing, supervision, project administration, and funding acquisition.

## Declaration of interests

M.W. received research support by Biogen in the past. C.G. reports that the University Hospital Basel, as the employer of C.G., has received the following fees, which were used exclusively for research support: (1) advisory board and consultancy fees from Actelion, Novartis, Genzyme-Sanofi, GeNeuro, Hoffmann La Roche, and Siemens; (2) speaker fees from Biogen, Hoffmann La Roche, Teva, Novartis, Merck, Jannsen Pharmaceuticals, and Genzyme-Sanofi; and (3) research grants from Biogen, Genzyme-Sanofi, Hoffmann La Roche, and GeNeuro.
