# Supplementary material for: Bridging mesoscopic and microscopic scales in multiple sclerosis: Post mortem brain block multi-contrast 9.4T MRI and histology quantification
Source: STAR Protoc. 2025 Nov 17;6(4):104203. doi: 10.1016/j.xpro.2025.104203 (PMC12666330; doi:10.1016/j.xpro.2025.104203)
Supplement: Document S1. Figures S1 and S2 [file mmc1.pdf]

STAR✦Protocols CellPress

Supplementary material

Bridging mesoscopic and microscopic scales in multiple sclerosis: 9.4T multi-contrast MRI of post-mortem brain blocks and histology quantification

Dimitrios G. Gkotsoulias[1,2,3,†,*], Lukas Schönenberger[1,2,3,†], Jochen Leupold[4,†], Ilaria Callegari[1,2,3], Erik Bahn[5], Christine Stadelmann[5], Dominik von Elverfeldt[4], Valerij G. Kiselev[4], Matthias Weigel[1,2,3,6] and Cristina Granziera[1,2,3,**]

[†]*These authors contributed equally*

[1] *Research Center for Clinical Neuroimmunology and Neuroscience Basel (RC2NB), University of Basel, CH*

[2] *Translational Imaging in Neurology Basel, Department of Biomedical Engineering, Faculty of Medicine, University of Basel, CH*

[3] *Department of Neurology, University Hospital Basel, CH*

[4] *Division of Medical Physics, Department of Diagnostic and Interventional Radiology, University Medical Center Freiburg, Faculty of Medicine, University of Freiburg, DE*

[5] *Department of Neuropathology, University Medical Center Göttingen, Göttingen, Germany*

[6] Division of Radiological Physics, Dept. of Radiology, University Hospital Basel, Basel, Switzerland

*Correspondence (Technical): dimitrios.gkotsoulias@unibas.ch

**Correspondence (Lead): cristina.granziera@unibas.ch

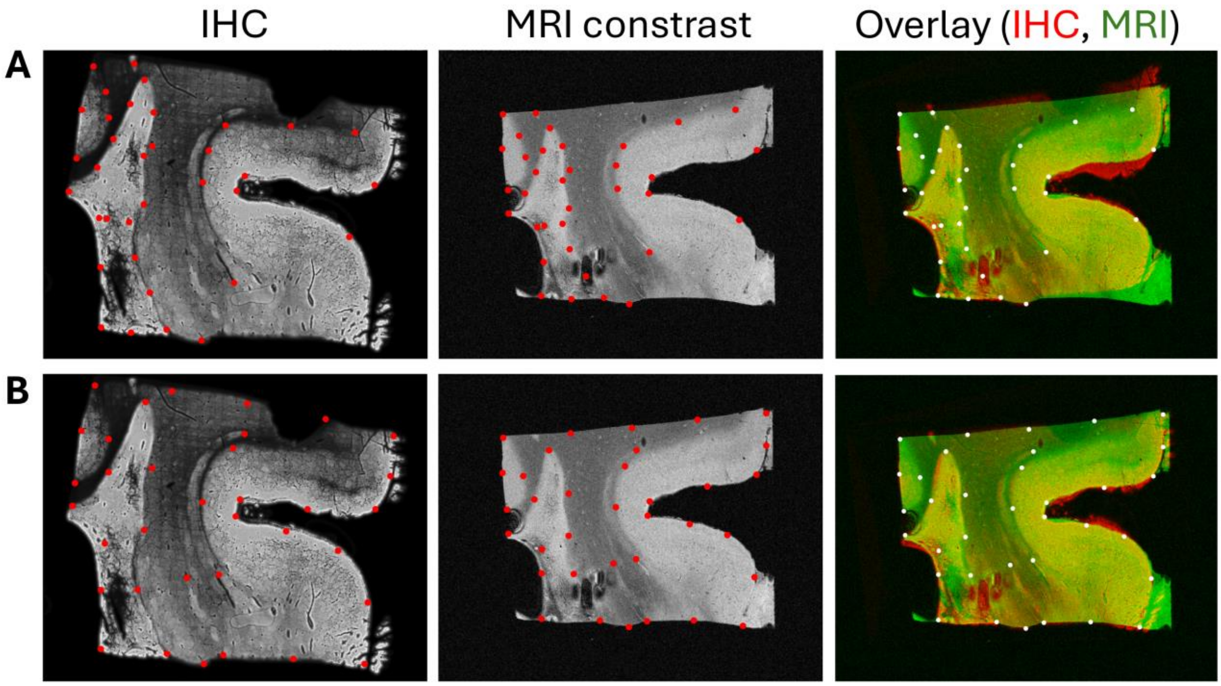

Supplementary Figure S1. Placement of landmarks for landmark-based non-linear registration steps between Immunohistochemistry (IHC) images and corresponding MRI contrast slices, related to Step '**Registration of histology-derived semi-quantitative maps, histological images and histology-derived segmentations with MRI contrasts****', Sub-step 40**: (**A**) Indicative suboptimal selection of landmarks and (**B**) optimal selection of landmarks allowing for significantly more overlap in the final registration between the 2 modalities.

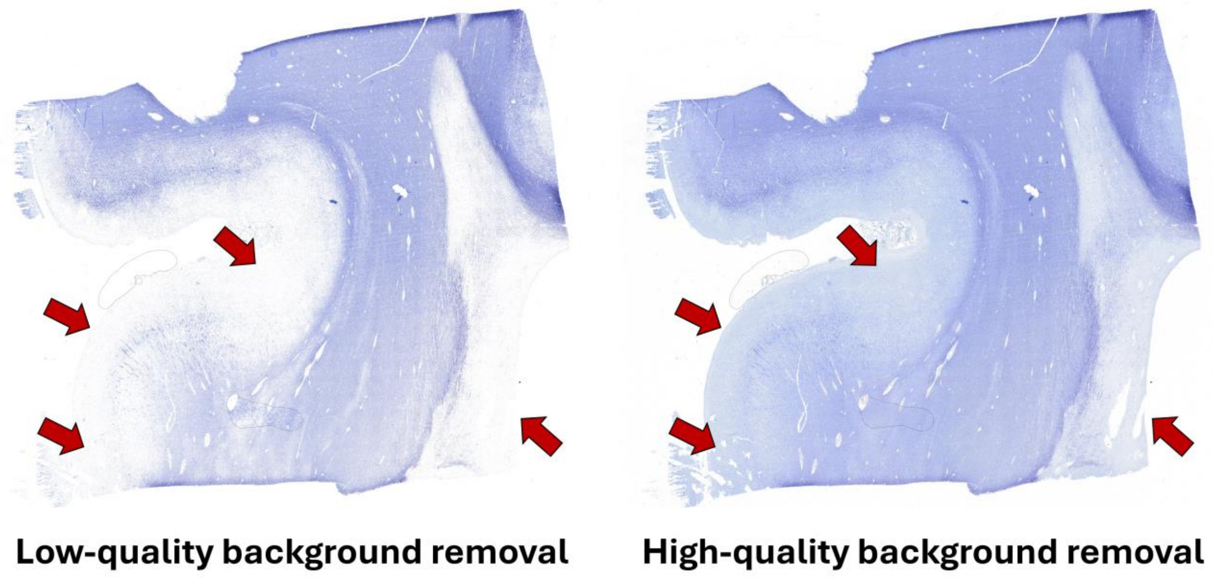

Supplementary Figure S2. Comparison between a suboptimal and an optimal case of background removal, associated with Troubleshooting Section, Problem 2: Suboptimal background removal can lead to ‘loss’ of tissue, in comparison to the optimal case. Relevant regions are indicated with red arrows.
